# Structural Covariance Network as an Endophenotype in Alzheimer’s Disease-Susceptible Single-Nucleotide Polymorphisms and the Correlations With Cognitive Outcomes

**DOI:** 10.3389/fnagi.2021.721217

**Published:** 2021-12-17

**Authors:** Hsin-I Chang, Yu-Tzu Chang, Chi-Wei Huang, Kuo-Lun Huang, Jung-Lung Hsu, Shih-Wei Hsu, Shih-Jen Tsai, Wen-Neng Chang, Chen-Chang Lee, Shu-Hua Huang, Chiung-Chih Chang

**Affiliations:** ^1^Department of Neurology, Cognition and Aging Center, Institute for Translational Research in Biomedicine, Kaohsiung Chang Gung Memorial Hospital, Chang Gung University College of Medicine, Kaohsiung, Taiwan; ^2^Division of Nephrology, Department of Internal Medicine, National Cheng Kung University Hospital, College of Medicine, National Cheng Kung University, Tainan, Taiwan; ^3^Department of Neurology, Chang Gung Memorial Hospital Linkou Medical Center and College of Medicine, Neuroscience Research Center, Chang Gung University, Taoyuan City, Taiwan; ^4^Department of Neurology, New Taipei Municipal TuCheng Hospital, New Taipei City, Taiwan; ^5^Graduate Institute of Mind, Brain, and Consciousness, Taipei and Brain and Consciousness Research Center, TMU Shuang Ho Hospital, Taipei Medical University, New Taipei City, Taiwan; ^6^Department of Radiology, Kaohsiung Chang Gung Memorial Hospital, Chang Gung University College of Medicine, Kaohsiung, Taiwan; ^7^Psychiatric Department of Taipei Veterans General Hospital, Taipei, Taiwan; ^8^Psychiatric Division, School of Medicine, National Yang-Ming University, Taipei City, Taiwan; ^9^Department of Nuclear Medicine, Kaohsiung Chang Gung Memorial Hospital, Chang Gung University College of Medicine, Kaohsiung, Taiwan

**Keywords:** Alzheimer’s disease, single nucleotide polymorphism, independent component analysis, apolipoprotein E4, structural covariance network, network integrity score

## Abstract

The cognitive manifestations of Alzheimer’s disease (AD) are related to brain network degeneration, and genetic differences may mediate network degeneration. Several AD-susceptible loci have been reported to involve amyloid or tau cascades; however, their relationships with gray matter (GM) volume and cognitive outcomes have yet to be established. We hypothesized that single-nucleotide polymorphism genotype groups may interact with apolipoprotein E4 (*ApoE4*) status or independently exert an effect on cognitive outcomes. We also hypothesized that GM structural covariance networks (SCNs) may serve as an endophenotype of the genetic effect, which, in turn, may be related to neurobehavior test scores. Gray matter SCNs were constructed in 324 patients with AD using T1 magnetic resonance imaging with independent component analysis (ICA). We assessed the effects of 15 genetic loci (rs9349407, rs3865444, rs670139, rs744373, rs3851179, rs11136000, rs3764650, rs610932, rs6887649, rs7849530, rs4866650, rs3765728, rs34011, rs6656401, and rs597668) using additive, recessive, and dominant models on cognitive outcomes. Statistical analysis was performed to explore the independent role of each locus, interactions with *ApoE4* status, and relationships to GM ICA network intensity score. For outcome measures, we used the Mini-Mental State Examination (MMSE), Cognitive Abilities Screening Instrument (CASI) total score, and short-term memory (STM) subscores, adjusted for the covariates of education, disease duration, and age. Clinically, the *CD2AP* G allele showed a protective role in MMSE, CASI total, and CASI-STM scores independently or via interactions with non-*ApoE4* status, while the *CR1* A genotype group was associated with lower STM subscores independent of *ApoE4* status. Three loci showed synergic interactions with *ApoE4*: *BIN 1, MS4A6A*, and *FTMT*. Of the 15 meaningful ICA components, 5 SCNs (anterior and posterior hippocampus, right temporal, left thalamus, default mode network) showed relationships with general cognitive performance, in which only the *ApoE4* and *MS4A6A* genotype groups were independently related to the hippocampus network. The genetic loci *MS4A6A, BIN1, CLU, CR1, BIN1, PICALM*, and *FGF1* influenced the networks independently or in synergy. This study suggests that AD-susceptible loci may each exert clinical significance independently through interactions with *ApoE4* status or through SCNs as an endophenotype and that this effect is associated with the cognitive outcomes.

## Background

The National Institutes on Aging-Alzheimer’s Association (NIA-AA) criteria (Jack et al., 2018) incorporate diagnostic biomarkers in the definition of Alzheimer’s disease (AD), and the key pathology is the presence of amyloid plaque and neurofibrillary tangles. From a genetic point of view, AD is a complex disease. For late-onset AD, the apolipoprotein E (ApoE) gene is the most significant example of a single-nucleotide polymorphism (SNP) that modifies susceptibility to AD (Singh et al., 2006). The clinical significance of *ApoE4* on brain integrity has been shown in studies of people with AD (Martins et al., 2005), those with mild cognitive impairment (Risacher et al., 2013), and the non-demented elderly (Bretsky et al., 2003; Wisdom et al., 2011). The presence of the *ApoE4* allele (Corder et al., 1993) remains the greatest risk factor for AD (Farrer et al., 1997), responsible for a 3- to 15-fold increase in risk. The synergic effect of the *ApoE4* allele with other genetic loci has been reported in previous studies (Mahley, 1988), which showed that overall pathological cascades may influence neuropsychiatric outcomes (Hall et al., 2014). Although genetic effects have been extensively studied, only a few studies have shown reproducible results. The mechanisms by which ApoE influences the pathogenesis of AD have been proposed, including a role in the clearance of amyloid (Koistinaho et al., 2004; Shi et al., 2017; Ringland et al., 2020). However, whether this is via interactions between APoE4 and amyloid- or tau-related SNPs remains to be explored.

Recent large-scale genome-wide association studies (GWASs) have revealed several SNPs other than *ApoE4* that are associated with susceptibility to AD. In a study by Lambert et al. (2013), a total of 19 GWAS loci were identified using a discovery sample of 17,008 patients with AD and 37,154 controls, followed by replication with 8,572 patients with AD and 11,312 controls. In 2019, a GWAS meta-analysis reported an additional 29 risk loci (Jansen et al., 2019). Collectively termed as AD-susceptible genes, they can only explain a small proportion of the heritability and influence of SNPs for risk prediction, and the results have been inconsistent among different ethnicities. For example, the rs11136000 polymorphism in the clusterin (*CLU*) gene has been significantly associated with AD (Zhu et al., 2018) in Caucasian and Asian populations, but not in people of African or Hispanic descent (Du et al., 2016). Genetic-gray matter (GM) interactions may be different among different ethnicities, and so the genetic effect on the cognitive outcomes may also be different. For AD, structural imaging is one of the most reliable tools to reveal correlations with cognitive measures, while links between reported genotype groups and neuroimaging biomarkers may help to understand how these SNPs modulate GM. The clinical significance of these risk loci and their interactions with brain networks may also help to understand the neurobiology of AD.

Recent research suggests that highly related regions show covariance in morphometric characteristics, the so-called structural covariance. Structural covariance networks (SCNs) have been associated with structural and functional connectivity, while genetic variations, developmental, degenerative, and disease staging have been shown to be important covariates of interest (Alexander-Bloch et al., 2013). SCNs are considered to be reproducible and heritable models that may represent disease-related or genetic-associated changes in topology (Huang et al., 2017; Chang et al., 2018a,b; Chang H. I. et al., 2019). We hypothesized that AD-susceptible genes may have greater covariance in GM and that the SCNs may serve as an endophenotype to reflect the influence of the risk gene. Using spatial independent component analysis (ICA), the structural covariance between topographically distant regions can be modeled without *a priori* knowledge (Biswal et al., 1995, 2010). In addition to fully automated spatial component maps of maximal statistical independence, a network integrity score (NIS) can be calculated for each ICA map. The NIS describes the strength of the individual expression in each network, and a higher NIS indicates a stronger individual expression of the identified network (Beckmann and Smith, 2004; Segall et al., 2012). Once an SCN pattern has been identified to distinguish one group from another, its expression can be prospectively quantified on an individual basis and correlated with the clinical or physiological measures of interest. GM SCNs modeled by ICA may help to clarify SNP-GM relationships, while the NIS can be used as a dependent variable to test the genetic interactions.

The NIA-AA criteria define AD as a disease status because higher diagnostic hierarchy is placed on the *in vivo* demonstration of neuropathological status rather than the presence of clinical symptoms. For a diagnosis of AD, the presence of amyloid and tau protein is essential (Jack et al., 2018). To understand the genetic effects of amyloid or tau on disease progression, we selected 15 amyloid- or tau-related SNPs belonging to the following genetic loci: *CD2AP* (rs9349407) (Furusawa et al., 2019), *CD33* (rs3865444) (Bradshaw et al., 2013; Chen et al., 2021), *MS4AE4* (rs670139) (Karch et al., 2012), *BIN1* (rs744373) (Martiskainen et al., 2015), *PICALM* (rs3851179) (Zhao et al., 2015), *CLU* (rs11136000) (Chibnik et al., 2011), *ABCA7* (rs3764650) (Bamji-Mirza et al., 2016; Zhao Q. F. et al., 2016; Ma et al., 2018), *MS4A6A* (rs610932) (Dos Santos et al., 2017), *FTMT* (rs6887649) (Yang et al., 2015), *SPTLC1* (rs7849530) (Geekiyanage and Chan, 2011), *Intergenic SNP* (rs4866650) (Hohman et al., 2014), p73 (rs3765728) (Wetzel et al., 2008), *FGF1* (rs34011) (Kimura et al., 1994), *CR1* (rs6656401) (Chibnik et al., 2011; Biffi et al., 2012), and *EXOC3L2* (rs597668) (Olgiati et al., 2011). In this study, we first tested whether each SNP exerted an effect on cognitive measures directly or via interactions with ApoE4. Then, we evaluated whether the genetic effects on cognitive measures were via an SCN as an endophenotype.

## Materials and Methods

This study was conducted in accordance with the Declaration of Helsinki and was approved by the Institutional Review Board of Chang Gung Memorial Hospital. The study participants were treated at the Cognition and Aging Center, Department of General Neurology, Kaohsiung Chang Gung Memorial Hospital. The multidisciplinary team was composed of behavioral neurologists, psychiatrists, neuropsychologists, neuroradiologists, and experts in nuclear medicine. We enrolled patients with AD who were diagnosed according to the International Working Group-2 criteria (Dubois et al., 2014) and further confirmed using amyloid imaging (TW-ADNI^1^) if the consensus panel did not agree on the diagnosis. All of the patients were in a stable condition under acetylcholine esterase inhibitor treatment from the time of diagnosis. The exclusion criteria were a history of clinical stroke, a negative amyloid scan, a modified Hachinski ischemic score > 4, and depression. After checking the inclusion and exclusion criteria, a total of 324 subjects (152 males and 172 females) were included and underwent imaging and genetic tests.

### Clinical and Neurobehavioral Assessments

After enrollment, the demographic data of each patient were recorded. We also recorded the time of the first symptom during interviews with family members so that an estimated duration of onset could be calculated. At baseline, a trained neuropsychologist administered the neurobehavioral tests using the Mini-Mental State Examination (MMSE) and Cognitive Abilities Screening Instrument (CASI) total score as a global assessment of cognitive function. The CASI contains nine subdomains. We used attention, verbal fluency, abstract thinking, and mental manipulation subdomain scores to assess executive function (Huang et al., 2013) and orientation, short- and long-term memory, language ability, and drawing as non-executive domains. As the salient feature of AD is short-term memory (STM) impairment, we used CASI-STM subscores as the major outcome for genetic correlations.

### Genotyping

Single-nucleotide polymorphism genotyping was performed using MassARRAY technology with iPLEX Gold chemistry (Agena Bioscience, San Diego, CA, United States). The PCR primers and single base extension primers were designed using Assay Design Suite version 2.0 software (Redwood City, CA, United States). The genotyping analysis was performed using an iPLEX Gold Reagent Kit according to the instructions of the manufacturer. Briefly, 1 μl of DNA sample (10 ng/μl) was added to 5 μl of PCR reaction mixture containing 0.2 units of Taq polymerase, 2.5 pmol each of the PCR primers, and 25 mM each of the dNTPs. Thermocycling was started at 94°C for 2 min followed by 45 cycles of 94°C for 30 s, 56°C for 30 s, and 72°C for 1 min, and a final extension was done at 72°C for 1 min. Unincorporated dNTPs were dephosphorylated using 0.3 U of shrimp alkaline phosphatase. Purified amplicons were then subjected to primer extension using an iPLEX Gold Reagent Kit. Primer extension was performed using a cycling program of 94°C for 30 s, followed by 40 cycles of 94°C for 5 s, and 5 cycles of 52°C for 5 s, and 80°C for 5 s within 40 cycles, followed by a final extension at 72°C for 3 min. The extended reaction products were purified using cation exchange resin and then spotted onto a 384-format SpectroCHIP II array using a MassArray Nanodispenser RS1000 (San Diego, CA, United States). Mass determination was done on a MassARRAY Compact Analyzer. The resulting spectra were processed, and alleles were called using MassARRAY Typer 4.0 with model-based cluster analysis to analyze the genotypes of the SNPs. We tested 15 SNPs (rs9349407, rs3865444, rs670139, rs744373, rs3851179, rs11136000, rs3764650, rs610932, rs6887649, rs7849530, rs4866650, rs3765728, rs34011, rs6656401, and rs597668). The risk alleles and minor allele frequencies (MAFs) are listed in Supplementary Table 1. The *ApoE* genotype was determined using rs7412 and rs429358. *ApoE4* carriers were defined as those with one or two E4 alleles.

### Image Acquisition

Magnetic resonance images were acquired using a 3.0T magnetic resonance imaging (MRI) scanner (Excite, GE Medical Systems, Milwaukee, WI, United States). All MRI images were performed within 3 months of taking the cognitive tests. High-resolution structural images were acquired for spatial normalization using the following protocol: a T1-weighted, inversion-recovery-prepared, three-dimensional, gradient-recalled acquisition in a steady-state sequence with a repetition time/echo time/inversion time of 8,600 ms/minimal/450 ms, a 256 mm × 256 mm field of view, and a 1-mm slice sagittal thickness with a resolution of 0.5 mm × 0.5 mm × 1 mm.

### Data Analysis for Neuroimaging Biomarkers

Image preprocessing and statistical analysis were performed using SPM12 (Wellcome Trust Centre of Cognitive Neurology, University College London, United Kingdom^2^). The T1 images were reoriented, realigned, and normalized using the standard Montreal Neurological Institute space. The images were then segmented into GM and white matter. Related tissue segments were used to create a custom template using diffeomorphic anatomical registration with an exponentiated lie algebra approach, which is one of the highest ranking registration methods in patients with AD (Cuingnet et al., 2011). To correct for partial volume effects, the segmentation of tissue type was performed using partial volume estimation. During the modulation step, each voxel of the registered GM image was multiplied by the Jacobian of the warp field that defined the direction and amount of modulation. The modulated and warped GM images were then smoothed using a Gaussian kernel of 8 mm full width at half maximum.

### Independent Component Analysis and Network Integrity Score

The preprocessed spatially normalized modified GM images of the patients were concatenated to form a subject series and entered into the ICA process. Spatial ICA was carried out using the Multivariate Exploratory Linear Optimized Decomposition into Independent Components software package version 3.15^3^. The resulting independent components were *z*-transformed and visualized using a threshold of *z* > 1.96 (*p* < 0.05). In general, the optimal number of components is related to the data size and the research questions. A lower number of components causes a loss of spatial information, while a higher number of components can result in less meaningful components. In this study, we set the number of independent components to 25. Among these 25 components, we determined 15 to be meaningful based on matching them with the published data and templates from a study investigating the social brain across childhood and adolescence (McCormick et al., 2018) and the BrainMap 70-component ICA template^4^.

### Statistical Analysis

We first tested whether each SNP exerted an effect on cognitive measures directly or via interactions (or synergistically) with *ApoE4*. Next, to evaluate whether the genetic effects on cognitive measures were via the SCN as an endophenotype, we explored the SCN-cognitive and SCN-SNP relationships. The SCN-cognitive relationships were explored using a linear regression model with the cognitive scores as the dependent variable and the NIS of each SCN as the independent variable, adjusted for age, education, and disease duration. Finally, we modeled how the 15 SNPs and *ApoE4* status may affect the GM SCN (and SCN-SNP relationship) using a multivariate linear model.

Clinical data were expressed as mean ± standard deviation. The Student’s *t*-test was used to compare continuous variables, and the chi-square test was used to compare categorical variables. To examine the genotype effects on cognitive outcome measures, we used additive, dominant, and recessive models (Zhao F. et al., 2016). The dichotomization of the SNP genotype into different genetic models was based on the risk allele. Based on the classification, the additive model compared three groups: risk homozygotes, risk heterozygotes, and non-risk homozygotes. For the dominant model, the genotype group was dichotomized into risk (homozygote and heterozygotes) and non-risk groups. For the recessive group, the risk homozygotes were considered to be one group and compared with the risk heterozygotes and non-risk homozygotes. For each SNP, the risk allele is listed in Supplementary Table 1 according to a literature review of the Chinese-Han population, and binary group stratification was based on the Hardy-Weinberg equation for equilibrium. Using the MMSE, CASI total score, or STM subscore as dependent variables, we also explored the interactions between the susceptible SNPs and ApoE4 status (ApoE4 carriers and non-E4 carriers) using a linear regression model, with education, age, and duration of disease as covariates. All statistical analyses were conducted using SPSS software (SPSS version 22 for Windows^®^, SPSS Inc., Chicago, IL, United States). Statistical significance was set at *p* < 0.05.

For each component, the intensity was calculated and recorded as NIS, which was then *Z*-transformed in the regression model. Differences in the ICA NIS of each SNP were calculated using the Student’s *t*-test based on the predefined groups in Supplementary Table 1. To understand the clinical significance of each SCN, we used a linear regression model to explore the relationships between outcome measures (MMSE, CASI total score, and executive and non-executive domains) and NIS with education, age, and duration of disease as covariates. In addition, to understand the clinical significance of the identified SCN in the CASI subscores, we also calculated partial correlations between the extracted SCN NIS and the cognitive scores by setting the significance value at *p* < 0.05 and the covariates as age, educational level, and disease duration (years).

Finally, multivariate linear regression analysis was performed to assess the SCN-SNP relationships. Fifteen models were run (one for each SCN NIS), and each model consisted of the following variables: age, sex, the 15 SNPs, and *APOE* E4 status. For the SNPs that showed significance on NIS, we tested the interactions with ApoE4 status. The NIS served as the dependent variable, with the significant SNP, ApoE4, and interaction (SNP*ApoE4) as independent variables. All independent variables were entered in one block and the significance value was set at *p* < 0.05.

## Results

### Demographic Data of the Patients With Alzheimer’s Disease

The demographic data and cognitive test scores are shown in Table 1. All of the enrolled patients had early-stage AD, as the mean duration of disease was 0.9 ± 1.12 years. Among the enrolled patients, 114 were E4 carriers (E4 homozygote *n* = 14). The risk alleles *CD2AP, ABCA7, FTMT, SPTLC1, FGF1, CR1*, and *EXOC3L2* were found to be minor alleles (Supplementary Table 1). Of note, the MAFs of *FTMT* and *CR1* were extremely low.

**TABLE 1:** Demographic data of the 324 patients with Alzheimer’s disease.

	Case numbers or scores
Male/Female	152/172
Education (year)	7.8 ± 5.09
Duration of disease (year)	0.9 ± 1.12
ApoE4 carriers (%)	114 (35.19%)
Age (years)	71.6 ± 8.8
Mini-mental state examination	21.1 ± 2.8
**Cognition Ability Screening Instrument**	
Total score (100)	68.6 ± 24.7
Executive function test (40)	25.5 ± 9.6
Attention (8) Verbal fluency (10) Abstract thinking (12) Mental manipulation (10)	6.3 ± 1.63 5.4 ± 3.08 8.1 ± 3.12 5.7 ± 3.50
Non-executive domains (60)	43.2 ± 15.83
Orientation (18) Short- term memory (12) Long-term memory (10) Language ability (10) Drawing (10)	13.0 ± 5.71 6.1 ± 4.11 8.3 ± 2.42 8.1 ± 2.42 7.7 ± 2.99

*Data presented as mean ± standard deviation. Numbers in parentheses indicate maximal score.*

For cognitive outcomes (MMSE, CASI total scores, and STM subscores), gender had no effect when controlling for educational level. For those with an educational level ≤ 6 years (elementary school), 7–12 years (high school), and >12 years, none of the comparisons of test scores between the male and female patients were significant. There were age effects on MMSE (*r* = − 0.23, *p* < 0.0001), CASI total (*r* = − 0.259, *p* < 0.0001), and STM subscores (*r* = − 0.271, *p* < 0.0001). The educational effects on MMSE (*r* = 0.432, *p* < 0.0001), CASI total (*r* = 0.433, *p* < 0.0001), and STM subscores (*r* = 0.372, *p* < 0.0001) were also significant.

### *CD2AP* and *CR1* on Cognitive Test Scores

The results of the additive model, dominant model, and recessive model of each SNP on MMSE and CASI are shown in Tables 2, 3. The *CD2AP* G allele was associated with MMSE total score (Table 2) or CASI total score (Table 3) in the additive or dominant model, and the G allele was associated with higher scores. The *CD2AP* G allele (in the additive or dominant model) was also associated with a higher STM subscore (Table 4). The association between the *CD2AP* G allele and cognitive scores showed interactions with the non-*ApoE4* genotype in MMSE (Table 2), CASI total score (Table 3), and STM subscore (Table 4). In the recessive model, the *CR1* A allele was associated with a lower STM subscore (Table 4), while the risk of a detrimental effect was independent of *ApoE4* status. Of note, the MAF of the A allele in *CR1* was 0.029.

**TABLE 2:** General linear model for single nucleotide polymorphisms on mini-mental score examination*.

Gene	Additive model				Dominant model			Recessive model			Interaction with APOE		
	Group	β	*SE*	*p*	β	*SE*	*p*	β	*SE*	*p*	Chi-square	*p*	effects
*CD2AP*	GG compares to CC (1/3)	9.180	3.8140	0.016	8.968	3.8115	0.019	–1.025	0.8284	0.216	8.065	0.026	Non-E4
rs9349407	CG compares to CC(2/3)	8.437	3.8528	0.029									
*CD33*	CC compares to AA (1/3)	1.810	2.0389	0.375	2.191	2.0287	0.280	0.875	0.7900	0.268	3.016	0.389	–
rs 3865444	CA compares to AA(2/3)	2.992	2.0962	0.153									
*MS4AE4*	GG compares to TT (1/3)	0.824	1.1392	0.470	1.054	1.0311	0.307	0.097	0.7921	0.903	3.252	0.354	–
s670139	GT compares to TT (2/3)	1.221	1.0883	0.262									
*BIN1*	AA compares to GG (1/3)	–0.399	1.2194	0.744	–0.043	1.1009	0.969	0.272	0.7868	0.729	2.559	0.465	–
rs744373	AG compares to GG(2/3)	0.055	1.1708	0.963									
*PICALM*	CC compares to TT (1/3)	–1.379	1.1078	0.213	–1.294	1.0134	0.202	0.485	0.7764	0.532	2.523	0.471	–
rs3851179	CT compares to TT (2/3)	–1.222	1.0822	0.259									
*CLU*	CC compares to TT (1/3)	–0.803	1.8222	0.660	–0.310	1.8107	0.864	1.373	0.7766	0.077	3.730	0.292	–
rs11136000	TC compares to TT (2/3)	0.650	1.8792	0.729									
*ABCA7*	TT compares to GG (1/3)	–0.371	1.1807	0.753	0.108	1.1082	0.922	0.808	0.7645	0.291	6.46	0.091	–
rs3764650	TG compares to GG(2/3)	0.570	1.1754	0.628									
*MS4A6A*	GG compares to TT (1/3)	–0.270	1.1356	0.806	0.461	1.0307	0.655	1.025	0.7877	0.193	5.518	0.138	–
rs610932	TG compares to TT (2/3)	0.986	1.0831	0.362									
*FTMT*	AA compares to GG (1/3)	–4.819	6.6353	0.468	–4.801	6.6335	0.469	0.206	1.0325	0.842	1.974	0.160	–
rs6887649	AG compares to GG(2/3)	–4.708	6.6912	0.482									
*SPTLC1*	AA compares to GG (1/3)	1.647	3.3356	0.622	1.465	3.3362	0.661	–1.030	0.9562	0.28	7.282	0.053	–
rs7849530	AG compares to GG(2/3)	0.661	3.4251	0.847									
*Intergenic SNP*	AA compares to CC (1/3)	–1.747	2.1482	0.416	–1.742	2.1286	0.670	0.161	0.7768	0.836	5.89	0.117	–
rs4866650	AC compares to CC(2/3)	–1.723	2.1886	0.428									
*P73*	GG compares to AA (1/3)	0.436	1.1243	0.698	0.456	0.7792	0.559	–0.201	1.0410	0.847	2.549	0.467	–
rs3765728	GA compares to AA(2/3)	0.463	0.8381	0.581									
*FGF1*	GG compares to AA (1/3)	–0.625	1.2399	0.671	–0.263	1.1872	0.825	0.576	0.7573	0.447	4.296	0.231	–
rs34011	GA compares to AA(2/3)	0.064	1.2681	0.960									
*CR1*	AA compares to GG (1/3)	–5.326	6.6206	0.421	5.231	6.6324	0.430	–2.032	1.6498	0.218	4.020	0.259	–
rs6656401	GA compares to GG(2/3)	–1.826	1.6971	0.282									
*EXOC3L2*	TT compares to CC (1/3)	0.138	1.1541	0.905	0.329	1.0693	0.758	0.239	0.7718	0.757	7.673	0.045	Non-E4
rs597668	TC compares to CC(2/3)	0.499	1.1362	0.661									

*Adjusted for education, disease duration and age. SE, standard error.

**TABLE 3:** General linear model for single nucleotide polymorphisms on cognitive ability screening instrument total scores*.

Gene	Additive model				Dominant model			Recessive model			Interaction with APOE		
	Group	β	*SE*	*p*	β	*SE*	*p*	β	*SE*	*p*	Chi-square	*p*	effects
*CD2AP*	GG compares to CC (1/3)	44.091	14.17	0.002	43.245	14.165	0.002	–4.337	3.0951	0.161	5.989	<0.05	Non-E4
rs9349407	CG compares to CC(2/3)	41.125	14.32	0.004									
*CD33*	CC compares to AA (1/3)	0.406	7.658	0.958	1.048	7.5987	0.890	1.746	2.9576	0.555	2.489	0.477	–
rs 3865444	CA compares to AA(2/3)	2.398	7.8728	0.761									
*MS4AE4*	GG compares to TT (1/3)	2.26	4.2595	0.596	4.152	3.8605	0.282	1.896	2.9644	0.522	6.002	0.112	–
s670139	GT compares to TT (2/3)	5.514	4.0691	0.175									
*BIN1*	AA compares to GG (1/3)	0.089	4.4880	0.984	1.080	4.1155	0.793	1.32	2.941	0.654	3.612	0.306	–
rs744373	AG compares to GG(2/3)	1.791	4.3096	0.678									
*PICALM*	CC compares to TT (1/3)	–3.254	4.1484	0.433	–2.431	3.7963	0.522	1.986	2.9024	0.494	3.117	0.374	–
rs3851179	CT compares to TT (2/3)	–1.733	4.0528	0.669									
*CLU*	CC compares to TT (1/3)	0.490	6.8294	0.943	1.801	6.7691	0.790	3.333	2.9119	0.252	2.972	0.396	–
rs11136000	TC compares to TT (2/3)	4.358	7.0434	0.536									
*ABCA7*	TT compares to GG (1/3)	0.127	4.4213	0.977	1.915	4.1498	0.644	2.657	2.8650	0.354	3.034	0.386	–
rs3764650	TG compares to GG(2/3)	3.636	4.4014	0.409									
*MS4A6A*	GG compares to TT (1/3)	–1.407	4.2567	0.741	0.329	3.8545	0.932	2.588	2.9492	0.380	4.623	0.202	–
rs610932	TG compares to TT (2/3)	1.562	4.0599	0.7									
*FTMT*	AA compares to GG (1/3)	26.705	24.7683	0.281	26.328	24.7763	0.288	2.905	3.8570	0.451	2.337	0.126	–
rs6887649	AG compares to GG(2/3)	24.296	24.9768	0.331									
*SPTLC1*	AA compares to GG (1/3)	11.770	12.4733	0.345	11.596	12.4562	0.352	1.663	3.5659	0.641	2.738	0.434	–
rs7849530	AG compares to GG(2/3)	10.829	12.8078	0.398									
*Intergenic SNP*	AA compares to CC (1/3)	–4.298	8.0343	0.593	–4.654	7.9625	0.559	–0.533	2.9044	0.854	3.990	0.263	–
rs4866650	AC compares to CC(2/3)	–5.279	8.1853	0.519									
*P73*	GG compares to AA (1/3)	–0.260	4.2196	0.951	1.091	2.9254	0.709	1.076	3.9070	0.783	3.319	0.345	–
rs3765728	GA compares to AA(2/3)	1.606	3.1456	0.610									
*FGF1*	GG compares to AA (1/3)	–6.776	4.5323	0.144	–5.592	4.4370	0.208	3.584	2.8329	0.206	5.267	0.153	–
rs34011	GA compares to AA(2/3)	–4.123	4.7375	0.384									
*CR1*	AA compares to GG (1/3)	34.971	24.7119	0.157	34.67	24.7429	0.161	–7.488	6.1687	0.225	3.999	0.135	–
rs6656401	GA compares to GG(2/3)	–5.771	6.3347	0.362									
*EXOC3L2*	TT compares to CC (1/3)	–2.806	4.3082	0.515	–4.447	3.9970	0.266	–1.65	2.8892	0.568	2.783	0.426	–
rs597668	TC compares to CC(2/3)	–5.899	4.2413	0.164									

*Adjusted for education, disease duration and age. SE, standard error.

**TABLE 4:** General linear model for single nucleotide polymorphisms on cognitive ability screening instrument short-term memory score*.

Gene	Additive model				Dominant model			Recessive Model			Interaction with APOE		
	Group	β	*SE*	*p*	β	*SE*	*p*	β	*SE*	*p*	Chi-square	*p*	Effects
*CD2AP*	GG compares to CC (1/3)	5.596	2.3663	0.018	5.418	2.3673	0.022	–0.790	0.5135	0.124	7.950	0.019	Non-E4
rs9349407	CG compares to CC(2/3)	4.971	2.3903	0.038									
*CD33*	CC compares to AA (1/3)	1.086	1.2685	0.392	1.225	1.2594	0.331	0.277	0.4910	0.573	5.597	0.133	–
rs 3865444	CA compares to AA(2/3)	1.518	1.3042	0.244									
*MS4AE4*	GG compares to TT (1/3)	0.506	0.7078	0.475	0.675	0.6407	0.292	0.095	0.4923	0.847	6.163	0.104	–
s670139	GT compares to TT (2/3)	0.797	0.6762	0.239									
*BIN1*	AA compares to GG (1/3)	0.488	0.7432	0.511	0.238	0.6819	0.727	–0.442	0.4869	0.364	7.7977	0.046	E4
rs744373	AG compares to GG(2/3)	0.059	0.7136	0.934									
*PICALM*	CC compares to TT (1/3)	0.035	0.6895	0.960	0.087	0.6308	0.890	0.061	0.4823	0.899	6.756	0.080	–
rs3851179	CT compares to TT (2/3)	0.132	0.6736	0.845									
*CLU*	CC compares to TT (1/3)	0.024	1.1313	0.983	0.268	1.1236	0.811	0.758	0.4824	0.116	6.739	0.081	–
rs11136000	TC compares to TT (2/3)	0.837	1.1667	0.473									
*ABCA7*	TT compares to GG (1/3)	1.112	0.7320	0.129	1.159	0.6856	0.091	–0.190	0.4760	0.690	6.606	0.086	–
rs3764650	TG compares to GG(2/3)	1.204	0.7287	0.098									
*MS4A6A*	GG compares to TT (1/3)	0.509	0.7056	0.470	–0.117	0.6389	0.855	0.632	0.4888	0.196	8.070	0.045	Non-E4
rs610932	TG compares to TT (2/3)	0.162	0.6730	0.809									
*FTMT*	AA compares to GG (1/3)	5.961	4.1087	0.147	–5.954	4.1075	0.147	0.161	0.6410	0.802	4.598	0.032	E4
rs6887649	AG compares to GG(2/3)	5.921	4.1433	0.153									
*SPTLC1*	AA compares to GG (1/3)	1.145	2.0743	0.581	1.198	2.0720	0.563	0.188	0.5928	0.751	6.861	0.076	–
rs7849530	AG compares to GG(2/3)	1.429	2.1299	0.502									
*Intergenic SNP*	AA compares to CC (1/3)	0.597	1.3297	0.653	–0.853	1.3220	0.519	–0.594	–0.4811	0.217	5.203	0.158	–
rs4866650	AC compares to CC(2/3)	1.302	1.3547	0.337									
*P73*	GG compares to AA (1/3)	0.545	0.7934	0.438	–0.424	0.4876	0.385	0.353	0.6516	0.588	6.753	0.080	–
rs3765728	GA compares to AA(2/3)	0.378	0.5244	0.471									
*FGF1*	GG compares to AA (1/3)	0.372	0.7702	0.629	–0.299	0.7369	0.685	0.212	0.4705	0.653	4.551	0.208	–
rs34011	GA compares to AA(2/3)	0.208	0.7877	0.792									
*CR1*	AA compares to GG (1/3)	5.587	4.0857	0.171	5.483	4.1096	0.182	–2.217	1.0189	0.030	4.670	0.198	–
rs6656401	GA compares to GG(2/3)	2.006	1.0473	0.055									
*EXOC3L2*	TT compares to CC (1/3)	0.131	0.7166	0.855	–0.115	0.6647	0.862	–0.382	0.4793	0.425	7.655	0.054	–
rs597668	TC compares to CC(2/3)	0.333	0.7055	0.637									

*Adjusted for education, disease duration, and age. SE, standard error.

### Effect of Interactions With the *ApoE4* Gene on Short-Term Memory Scores

Short-term memory is the salient feature in AD, and we found three additional SNPs that showed interactions with the *ApoE4* gene: *BIN 1, MS4A6A*, and *FTMT* (Table 4 and Supplementary Figure 1). The E4 allele was related to lower scores in the *BIN 1, FTMT*, and *MS4A6A* alleles. The MAF of the G allele in *FTMT* was 0.082.

### Network Spatial Topography and Cognitive Significance Validation

**FIGURE 1:**
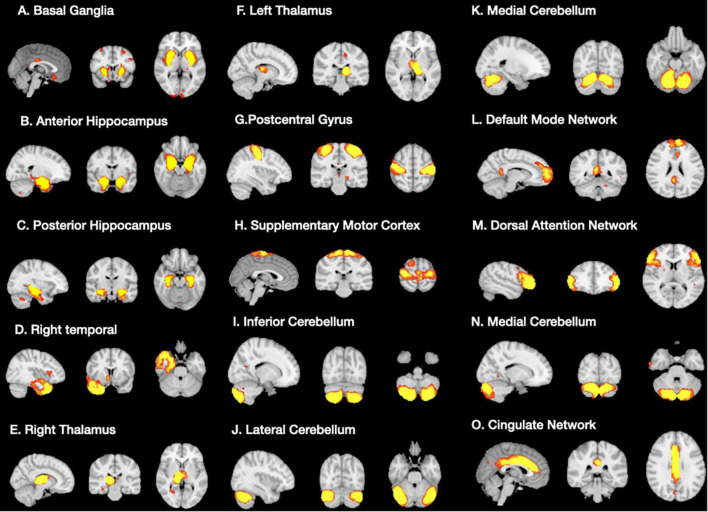
Significant structural covariance network **(A–O)** showing group differences in signal intensities using independent component analysis.

**FIGURE 2:**
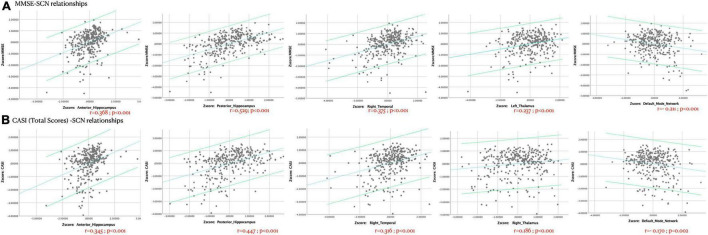
The *Z*-transformed cognitive **(A)** Mini-Mental State Examination [MMSE] or **(B)** Cognitive Ability Screening Instrument [CASI]-structural covariance network relationships, adjusted for age, years of education, and disease duration (years). Dots represent each participant in this study, and the linear relationships and 95% confidence intervals of individuals are shown. *r* = partial correlation coefficient. The exact *p*-values are shown.

A total of 15 ICA components were constructed and considered to be meaningful networks (Figure 1). The MMSE and CASI total scores were considered to indicate general cognitive performance, and their relationships with significant NIS are shown in Figure 2. Among the significant networks, the anterior and posterior hippocampus, right temporal, left thalamus, and default mode network were the most important for general cognitive performance. We then used NIS as the dependent variable to explore its relationships with STM, executive domains, and non-executive domains, adjusted for age and educational level. The standardized beta coefficients (95% confidence intervals) and exact *p*-values are reported in Table 5. For STM, executive domains, and non-executive domains, the aforementioned five networks (Figure 2) showed statistical significance, while the dorsal attentional network also showed statistical significance. Finally, we examined the relationships between CASI subdomains and NIS (Supplementary Table 2). The results suggested that the NIS of the anterior and posterior hippocampus, right temporal, right thalamus, lateral cerebellum, and medial cerebellum (all *p* < 0.0001) were consistently related to the cognitive test scores adjusted for age and years of education.

**TABLE 5:** Linear regression model between network intensity score analysis and cognitive test scores (Z transformed).

Area		Unstandardized beta; 95% Confidence Interval (*p*-value)		

Index on Figure 1	Network main structure	Short term memory score	Non-executive score	Executive score
A	Basal ganglia	–0.312; –0.749 ∼0.116 (*p* = 0.153)	–0.343; –1.984 ∼1.297 (*p* = 0.681)	–0.533; –1.5 ∼0.433 (*p* = 0.278)
B	Anterior hippocampus	1.518; 1.102∼1.934 (*p* < 0.001)	6.064; 4.486∼7.642 (*p* < 0.001)	2.118; 1.134∼3.102 (*p* < 0.001)
C	Posterior hippocampus	2.125; 1.717∼2.533 (*p* < 0.001)	7.691; 6.105∼9.278 (*p* < 0.001)	3.415; 2.422∼4.407 (*p* < 0.001)
D	Right temporal	1.292; 0.872 ∼1.712 (*p* < 0.001)	5.045; 3.444 ∼6.646 (*p* < 0.001)	2.384; 1.419∼3.350 (*p* < 0.001)
E	Right thalamus	0.411; –0.022∼0.844 (*p* = 0.063)	2.037; –0.388∼3.685 (*p* = 0.016)	1.219; 0.246∼2.191 (*p* = 0.014)
F	Left thalamus	0.591; 0.164∼1.017 (*p* = 0.007)	2.354; 0.724∼3.984 (*p* = 0.005)	1.525; 0.566∼2.484 (*p* = 0.002)
G	Postcentral gyrus	–0.477; –0.896∼–0.058 (*p* = 0.026)	–1.447; –3.054∼–0.151 (*p* = 0.078)	–0.409; –1.361∼0.543 (*p* = 0.398)
H	Supplementary motor cortex	–0.439; –0.860∼–0.019 (*p* = 0.041)	–1.130; –2.743∼0.484 (*p* = 0.169)	–0.685; –1.637∼0.267 (*p* = 0.158)
I	Inferior cerebellum	–0.442; –0.859∼–0.026 (*p* = 0.037)	–1.917; –3.505∼–0.329 (*p* = 0.018)	–0.729; –1.671∼0.214 (*p* = 0.129)
J	Lateral cerebellum	–0.181; –0.613∼0.251 (*p* = 0.410)	–0.752; –2.404∼0.9 (*p* = 0.371)	–0.034; –1.010∼0.942 (*p* = 0.945)
K	Medial cerebellum	–0.426; –0.853∼0.001 (*p* = 0.05)	–2.595; –4.212∼–0.977 (*p* = 0.002)	–1.262; –2.221∼–0.303 (*p* = 0.01)
L	Default mode network	0.668; 0.253∼1.083 (*p* = 0.002)	1.263; 0.296∼2.177 (*p* = 0.01)	2.582; 1.001∼4.170 (*p* = 0.001)
M	Dorsal attention network	0.682; 0.273∼1.092 (*p* = 0.001)	3.119; 1.566∼4.672 (*p* = 0.001)	1.956; 1.042∼2.869 (*p* = 0.001)
N	Medial cerebellum	–0.073; –0.493 ∼0.346 (*p* = 0.731)	–0.472; –2.075∼1.132 (*p* = 0.563)	–0.635; –1.579∼0.31 (*p* = 0.187)
O	Cingulate network	–0.085; –0.333∼0.504 (*p* = 0.688)	0.567; –1.033∼2.168 (*p* = 0.486)	–0.398; –0.546∼1.3343 (*p* = 0.407)

### Structural Covariance Networks–Single-Nucleotide Polymorphism Relationships

**FIGURE 3:**
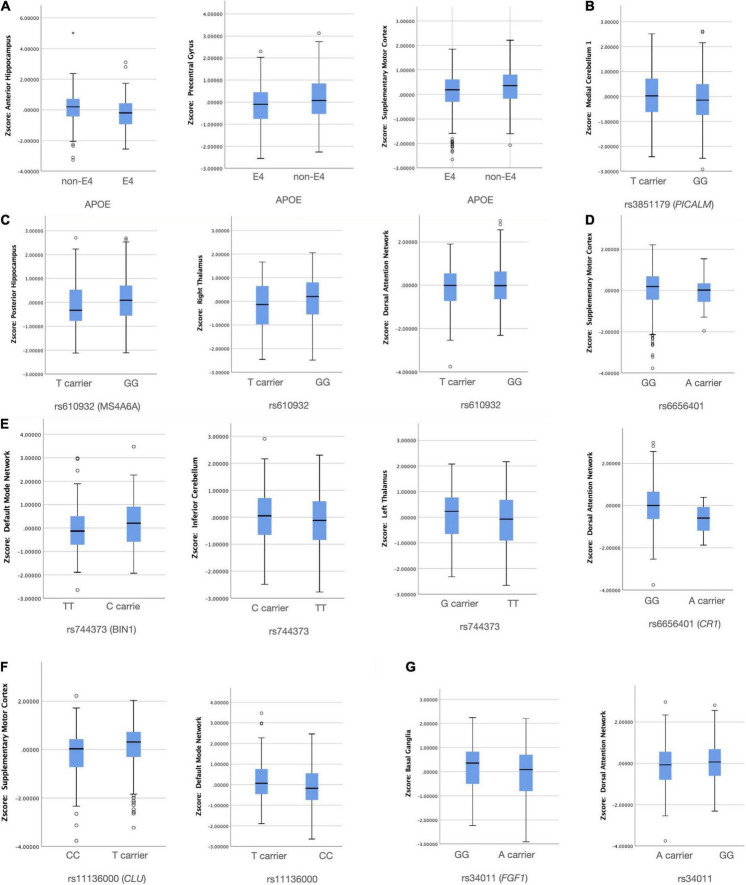
Significant group differences between the risk and non-risk allele **(A–G)** in network intensity score (*p* < 0.05). *X* axis: SNP group, *Y* axis: Z transformed network intensity values.

Using the binary classifications as described in Supplementary Table 1, seven SNPs were found to have an independent effect on NIS (Figure 3). To avoid possible false positives based on the 16 × 15 SCN-SNP matrix and a *p*-value of 0.05, we further explored the SCN-SNP relationships (Table 6) using a multivariate linear model. The NIS served as the dependent variable, and the independent variables included ApoE4 status, rs9349407, rs3865444, rs670139, rs744373, rs3851179, rs11136000, rs3764650, rs610932, rs6887649, rs7849530, rs4866650, rs3765728, rs34011, rs6656401, and rs597668, adjusted for age and sex. Among the tested 16 genetic loci, the significance of the 7 SNPs (*ApoE4, MS4A6A, BIN1, CLU, CR1, PICALM*, and *FGF1*) showed independent or collinear relationships with the SCNs. The predictive roles of *CLU* and *CR1* on supplementary motor cortex SCNs were independent, and no interactions with ApoE4 were found.

**TABLE 6:** Risk allele per structural covariance network of 15 tested single nucleotide polymorphisms (SNP) and ApoE4.

Network	SNP	Risk allele	Unstandardized B (95%CI)	Standardized β	*P*-value
Basal ganglia	*FGF1* (rs34011)	A-carrier	–0.261(–0.026∼–0.497)	–0.142	0.03
Anterior Hippocampus	*ApoE4*	E4 carrier	–0.344 (–0.619∼–0.069)	–0.164	0.014
posterior Hippocampus	*MS4A6A* (rs610932)	T carrier	0.292(0.024∼0.559)	0.154	0.033
Right Thalamus	*MS4A6A* (rs610932)	T carrier	0.417 (0.121∼0.712)	0.199	0.006
Left Thalamus	*BIN1* (rs744373)	TT	–0.294(–0.557∼–0.031)	–0.145	0.028
Precentral Gyrus	*APOE4*	E4	0.421 (0.173–0.669)	0.217	0.001
Supplementary Motor Cortex	*APOE4*	E4 carrier	0.302(0.074∼0.53)	0.169	0.01
	*CLU (rs11136000)*	CC	0.258(0.032∼0.484)	0.143	0.025
	*CR1* (rs6656401)	A carrier	–0.482(—-0.954∼–0.011)	–0.130	0.045
Inferior Cerebellum	*BIN1* (rs744373)	TT	–0.335(–0.607∼–0.062)	–0.161	0.016
Medial Cerebellum	*PICALM (rs3851179)*	GG	–0.266 (–0.521∼–0.011)	–0.135	0.041
Default Mode Network	*CLU* (rs11136000)	CC	–0.313(–0.566∼–0.06)	–0.157	0.016
	*BIN1* (rs744373)	TT	0.320(0.063∼0.576)	0.161	0.015
Dorsal Attention Network	*CR1* (rs6656401)	A-carrier	–0.640(–1.184∼–0.096)	0.103	0.021
	*FGF1* (rs34011)	A-carrier	0.274(0.025∼0.255)	0.137	0.031
	*MS4A6A* (rs610932)	T carrier	0.364(0.076∼0.652)	0.175	0.014

*The Multivariate linear model was used. Independent variables using ApoE4, rs9349407, rs3865444, rs670139, rs744373, rs3851179, rs11136000, rs3764650, rs610932, rs6887649, rs7849530, rs4866650, rs3765728, rs34011, rs6656401, rs597668, adjusted for age and gender; dependent variable is the network integrity score.*

## Discussion

### Major Findings

In this study, we tested the effects of amyloid- or tau-related genotypes on cognitive measurements and evaluated whether the genetic effects on cognitive measures were via SCNs as an endophenotype. We found that three different mechanisms modulated the relationships among AD-susceptible loci, GM network, and cognitive measures. First, an independent role of the genetic effect on cognitive test scores was found, as the *CD2AP* and *CR1* genotypes were directly related to CASI and MMSE scores. Second, the effects of amyloid- or tau-related genotypes on cognitive measures interacted or had a synergic effect with *ApoE4* status. Finally, we identified several SCNs that may serve as endophenotypes of AD-susceptible loci in predicting cognitive outcomes. The SCNs showing clinical significance with cognitive scores included the hippocampal, temporal, thalamus, default mode network, and dorsal attention network. In multivariate regression, seven genotype groups were found to either exert an independent effect or synergistic effect on these SCNs. As the SCNs were associated with different cognitive domains, the identification of genotype-SCN relationships may help to understand the neurobiology of SNPs on the cognitive features in AD.

### Dominant Model Suggested Protection of the *CD2AP* rs9349407 G Allele in Non-E4 Carriers

The role of rs9349407 as a risk SNP in Han Chinese populations has been reported (Xiao et al., 2015); however, the findings have been inconsistent (Jiao et al., 2015). One meta-analysis suggested that rs9349407 C is a risk allele for susceptibility to AD in East Asian, American, Canadian, and European populations (Chen et al., 2015). In our additive and dominant models, patients with AD with the rs9349407 G allele had higher CASI total and STM subscores, especially the non-E4 carriers. The minor allele C of rs9349407 has been associated with neuritic plaque burden, which may explain why non-E4 carriers with the G allele may have higher cognitive test scores (Shulman et al., 2013).

In this study, we only enrolled patients with clinical AD, and we tested whether risk SNP alleles or protective SNP alleles affected cognitive test scores or SCNs. Although not all of our patients with AD received amyloid scans, our exclusion criteria reduced the possibility of non-AD pathologies. Rs9349407 is a polymorphism in the *CD2AP* gene that translates the scaffolding molecule for signal transduction. Loss of function of *CD2AP* has been linked to enhanced Aβ production, tau-induced neurotoxicity, abnormal neurite structure modulation, and reduced blood-brain barrier integrity, which has been implicated in AD pathogenesis (Dubey et al., 2018; Ramos de Matos et al., 2018).

### *CR1* rs6656401 A Allele on Cognitive Outcomes

*CR1* is an AD susceptibility locus that also influences AD-related traits on neuritic plaque deposition and episodic memory decline. As STM is the salient feature in AD, it was used in this study as a dependent variable in the SNP genetic model analysis. We identified the independent role of the rs6656401 A allele on lower STM subscores, consistent with a previous meta-analysis of greater risk with the minor allele A in AD (Shen et al., 2015). A coding variant in the long homologous repeat D region of the *CR1* gene, rs4844609 (Ser1610Thr), has been associated with episodic memory decline and been shown to account for the known effect of SNP rs6656401 (Keenan et al., 2012). *CR1* risk allele A was a minor allele with a low MAF in this study, and the relationship with lower STM may be due to the small number of patients with the A allele. However, three studies have confirmed the association between AD susceptibility and the rs6656401 A allele in Chinese patients (Zhang et al., 2010; Chen et al., 2012; Jin et al., 2012), although another study showed no association (Li et al., 2011). Rs6656401 is in the *CR1* gene, and complement system activation in the clearance of amyloid has been proposed to be a possible mechanism of the risk associated with rs6656401 (Zhang et al., 2010). Although the independent role of rs6656401 minor allele on lower STM was confirmed in this study, it is worth noting that the A allele was a minor allele and the MAF was only 0.029. Therefore, the findings related to *CR1* in this study should be interpreted with caution.

To understand the effect, we also evaluated SCN intensity and explored whether the relationships between the rs6656401 A allele and cognitive test results may have been modulated by SCNs. As shown in our regression model, the dorsal attention SCN intensity and supplementary motor cortex SCN were significantly different between the risk and protective alleles, and the network intensity was also related to the executive and non-executive domains. Therefore, we suggest that the dorsal attention network is the endophenotype of the link between rs6656401 and cognitive outcomes.

### Structural Covariance Networks as an Endophenotype Between Risk Single-Nucleotide Polymorphism and Cognitive Test Scores

Based on the relationships between SCN and cognitive test scores, we identified several significant networks that predicted general or isolated cognitive function in the patients (Table 5, Figure 2, and Supplementary Table 2). For these significant networks, we identified independent or synergic effects in seven genotype groups that predicted the network integrity. As the clinical significance of SCNs was established in regression analysis with different cognitive domains, SCNs may have served as the endophenotype between the risk SNP group and cognitive test scores.

### *ApoE4* Modulated Anterior Hippocampal, Precentral Gyrus, Supplementary Motor Cortex and Determined the Salient Features of Alzheimer’s Disease

By definition, SCNs are based on the similarity of the same microstructural variations and thus may be influenced by factors influencing underlying structures such as the expression of common genetic traits during development. The independent role of *ApoE4* on the anterior hippocampus and precentral and supplementary motor cortex SCN was established in this study. As the hippocampal SCN intensity scores were also correlated with STM subscores in our patients with AD, this finding supports the role of *ApoE4* in linking salient cognitive and biosignature features in AD.

An important finding in this study is the relationship between *ApoE4* and the anterior hippocampal area. Traditional cytoarchitectonic distribution of the hippocampus (Frederickson et al., 1983) is a convoluted GM structure encompassing three architectonically distinct regions: the fascia dentata, the CA region (which can be subdivided into CA1–CA4 fields), and the subicular complex. In this study, two hippocampus SCNs in ICA (anterior vs. posterior hippocampus) showed *ApoE4* genotype group differences. The SCN of the hippocampal axis associated with *ApoE4* showing the anterior-posterior axis is consistent with task-related activities or connectivity patterns (Colombo et al., 1998; Przeździk et al., 2019). A similar anterior-posterior organization has recently been reported, suggesting that the gene expression is linked to the distinct molecular gradient in the hippocampus (Vogel et al., 2020). Among the 15 preselected SNPs and *ApoE4*, *ApoE4* had an independent role on the anterior hippocampal axis, in contrast to *MS4A6A* on the posterior axis. The association with cognitive test scores in hippocampal SCN intensity scores and the interactions between the hippocampus and cortical/subcortical structures demonstrate its close integration within large-scale degenerative systems.

### *MS4A6A* Modulated Posterior Hippocampus, Right Thalamus, and Dorsal Attention Networks

A previous study reported interactions between *MS4A* and *CLU* (Lambert et al., 2013) or *CD33* on conferring the risk of AD. Meanwhile, common variants of *MS4A6A* (rs610932), *MS4A4E* (rs670139), *CD33* (rs3865444), *CD2AP* (rs9349407), *CLU*, and *PICALM* have been associated with memory decline (Hollingworth et al., 2011; Naj et al., 2011; Karch et al., 2012). These reports emphasize the risk and role of *MS4A6A* in disease progression, while our results emphasize the role of *MS4A6A* (rs610932) and posterior hippocampus in the clinical features of AD. Similar to a report with a smaller AD cohort (Chang Y. T. et al., 2019), the dissociation of the hippocampus (anterior or posterior) emphasizes the interplay of genetic loci on anatomy.

### *BIN1* and *CLU* Modulated Default Mode Network Structural Covariance Networks

The most commonly affected brain region in AD studies is the default mode network, and the deposition of amyloid or tau protein is localized in this network (Hansson et al., 2017; Jones et al., 2017). The default mode network is regarded to be an early neuroimaging biosignature (Chang et al., 2015), and a recent report suggested that the default mode network may be comprised of multiple, spatially dissociated but interactive components (Andrews-Hanna et al., 2010), of which two subsystems are of particular interest. Our SCN results of associations with *BIN1* and *CLU* are consistent with cores in the posterior cingulate cortex and anterior medial prefrontal cortex, which is known as the “dorsal medial prefrontal cortex subsystem” (or midline core subsystem). The genetic locus rs744373 near *BIN1* (OR = 1.13; 95% CI, 1.06–1.21 per copy of the minor allele; *p* = 1.59 × 10^–11^) has been related to AD, with the possible mechanism involving tau-related cascade (Franzmeier et al., 2019). *BIN1* also modulated the inferior cerebellum and left thalamus and could predict cognitive scores in this study.

### Limitations and Methodological Considerations

This study has several limitations. First, we enrolled subjects with early-stage AD, and we did not include a control group or subjects with late-stage AD. As the SNPs were preselected from GWAS results showing significance in AD susceptibility, the inclusion of a control group may have helped to elucidate whether these SNPs exerted similar GM modulation patterns in healthy elderly subjects as in those with AD. However, as these SNPs each exert different functional activities on the pathogenetic mechanisms in AD, the use of a pure AD population may help to maximize the effect of each SNP on regional GM networks. As a structural covariance matrix is defined by estimating the interregional correlations of cortical volumes between all possible pairs of regions defined by anatomy, SCN construction relies on both the spatial patterns of morphometric and signal similarities. Given the differences in brain morphometry in controls, it would be difficult to match all influential factors. In this study, we did not include a late-stage AD group based on two considerations. First, we wanted to examine interactions between SCNs and SNPs in a more uniform population, so only subjects with early-stage AD were included. Second, the enrollment of subjects with late-stage AD may have included SCNs related to disease progression; however, this was not the major purpose of this study. The effects of susceptible SNPs and disease progression are important scientific questions. However, we focused on the initial hypothesis and tested the endophenotypic role of SCNs in cognitive outcomes in a uniform population with early-stage AD. A larger sample size including subjects with different stages of AD may help to extend the understanding of susceptible SNPs and brain degeneration patterns.

Another limitation is the estimation of the number of components for ICA analysis. Most studies have used 12–30 components in structural networks or resting-state networks. In this study, we constructed SCN networks and filtered the clinical significance of a network using correlation analysis with cognitive measures and by matching with the ICA template (see footnote 4). From a methodological aspect, the groupwise structural covariance analysis relied on the morphological properties of each voxel with the rest of the brain across a group of participants. The registration of the structural data of a single participant to the template involves linear and non-linear deformation that can result in inaccuracies in subregions. In this report, we emphasized well-characterized networks to explain the SNP effect, where the network significance was established by correlations with cognitive measures. Finally, the use of 15 AD-susceptible SNPs to validate the underlying pathological mechanisms may have oversimplified the genetic interactions. However, these SNPs were chosen based on their significance in large-scale GWASs in which they were all involved in amyloid or tau cascades, so the hypothesis was to test their effect on cognitive scores. We only tested interactions between each SNP and *ApoE4* status, as *ApoE4* remains the strongest predictor. The interpretations of interactions between SNPs and SCNs were based on a literature review of possible alterations in functional pathways and may not fully explain the *in vivo* situation. Therefore, the findings should be interpreted with caution.

## Conclusion and Future Perspectives

In AD, our findings demonstrated amyloid or tau-related SNP effects that may influence SCNs independently or synergistically with *ApoE4*. The use of SCNs as an endophenotype allowed us to assume the independent and synergistic role of putative SNPs to predict cognitive measures. The complex interplay among these SNPs in our study suggests that the hierarchical order of SNPs modulates GM networks.

## Data Availability Statement

The raw data supporting the conclusions of this article will be made available by the authors, without undue reservation.

## Ethics Statement

The studies involving human participants were conducted in accordance with the Declaration of Helsinki and were reviewed and approved by the Institutional Review Board of Chang Gung Memorial Hospital. Written informed consent to participate in this study was provided by the participants.

## Author Contributions

C-WH, K-LH, J-LH, S-WH, S-JT, W-NC, C-CL, S-HH, and C-CC contributed to the acquisition and analysis of data. H-IC, Y-TC, and C-CC drafted a significant portion of the manuscript and figures. All authors read and approved the final manuscript.

## Conflict of Interest

The authors declare that the research was conducted in the absence of any commercial or financial relationships that could be construed as a potential conflict of interest. The reviewer K-JT declared a shared affiliation, with no collaboration, with one of the authors, Y-TC, to the handling editor at the time of the review.

## Publisher’s Note

All claims expressed in this article are solely those of the authors and do not necessarily represent those of their affiliated organizations, or those of the publisher, the editors and the reviewers. Any product that may be evaluated in this article, or claim that may be made by its manufacturer, is not guaranteed or endorsed by the publisher.

## Footnotes

1
http://tadni.cgmh-mi.com/home

2
http://www.fil.ion.ucl.ac.uk/spm/

3
http://fsl.fmrib.ox.ac.uk/fsl/fslwiki/MELODIC

4
https://brainmap.org/icns/

## Supplementary Material

Supplementary Figure 1 Effect of interactions with the *ApoE4* gene on STM scores was found in *CD2AP*, *BIN 1, MS4A6A*, and *FTMT*.
