# Supplementary material for: Structural Covariance Network as an Endophenotype in Alzheimer’s Disease-Susceptible Single-Nucleotide Polymorphisms and the Correlations With Cognitive Outcomes
Source: Front Aging Neurosci. 2021 Dec 17;13:721217. doi: 10.3389/fnagi.2021.721217 (PMC8719443; doi:10.3389/fnagi.2021.721217)
Supplement: Supplementary file 1 [file Table_1.docx]

Supplementary Table 1. Fifteen single nucleotide polymorphisms (SNPs) and allele frequencies of 324 Alzheimer disease patients

| Gene name | SNP | Chromosome | A1 | A2 | MAF | Group stratification | Risk (protective) allele from reference |
| --- | --- | --- | --- | --- | --- | --- | --- |
| *CD2AP* | rs9349407 | 6 | C | G | 0.3606 | C carrier (n=99)/GG (n=225) | C [1] |
| *CD33* | rs3865444 | 19 | A | C | 0.20766773 | A carrier (n=90)/CC (n=234) | (T carrier) [2, 3] |
| *MS4AE4* | rs670139 | 11 | T | C | 0.40353698 | T carrier (n=214)/GG (n=110) | C [4] |
| *BIN1* | rs744373 | 2 | G | T | 0.38782051 | G carrier (n=124)/ AA (n=200) | T [2] |
| *PICALM* | rs3851179 | 11 | A | G | 0.39262821 | T carrier (n=130)/CC (n=194) | G [5] [6] [7, 8] |
| *CLU* | rs11136000 | 8 | T | C | 0.22829582 | T carrier (n=114)/CC (n=210) | (T carrier) [9] [10] |
| *ABCA7* | rs3764650 | 19 | G | T | 0.3538961 | G carrier (n=177)/TT (n=147) | G [11] |
| *MS4A6A* | rs610932 | 11 | T | G | 0.4057508 | T carrier (n=209)/GG (n=115) | (T Carrier) [3] |
| *FTMT* | rs6887649 | 5 | G | A | 0.08173077 | G carrier (n=50)/AA(n=274) | G [12] |
| *SPTLC1* | rs7849530 | 9 | G | A | 0.10289389 | G carrier (n=60)/AA (n=264) | G [12] |
| *Intergenic SNP* | rs4866650 | 5 | C | A | 0.20512821 | C carrier (n=118)/AA (n=206) | C [12] |
| *P73* | rs3765728 | 1 | A | G | 0.62666667 | G carrier (n=176)/AA (n=148) | G [13] |
| *FGF1* | rs34011 | 5 | A | G | 0.31331169 | A carrier (n=157)/GG (n=167) | A [13] |
| *CR1* | rs6656401 | 1 | A | G | 0.02884615 | A carrier (n=17)/GG (n=307) | A [7] [9] |
| *EXOC3L2* | rs597668 | 19 | C | T | 0.37299035 | C carrier (n=187)/TT (n=137) | C [4] |

Risk alleles according to the sited references, representing significant differences between controls and Alzheimer disease patients.

A1=minor allele; A2=major allele; MAF=minor allele frequency in this study

**Literature review focused on Asian populations**

1. Xiao, Q., et al., *Risk prediction for sporadic Alzheimer's disease using genetic risk score in the Han Chinese population.* Oncotarget, 2015. **6**(35): p. 36955-64.

2. Tan, L., et al., *Association of GWAS-linked loci with late-onset Alzheimer's disease in a northern Han Chinese population.* Alzheimers Dement, 2013. **9**(5): p. 546-53.

3. Deng, Y.L., et al., *The prevalence of CD33 and MS4A6A variant in Chinese Han population with Alzheimer's disease.* Hum Genet, 2012. **131**(7): p. 1245-9.

4. Jiao, B., et al., *Polygenic Analysis of Late-Onset Alzheimer's Disease from Mainland China.* PLoS One, 2015. **10**(12): p. e0144898.

5. Li, H.L., et al., *PICALM and CR1 variants are not associated with sporadic Alzheimer's disease in Chinese patients.* J Alzheimers Dis, 2011. **25**(1): p. 111-7.

6. Yu, J.T., et al., *Genetic association of PICALM polymorphisms with Alzheimer's disease in Han Chinese.* J Neurol Sci, 2011. **300**(1-2): p. 78-80.

7. Chen, L.H., et al., *Polymorphisms of CR1, CLU and PICALM confer susceptibility of Alzheimer's disease in a southern Chinese population.* Neurobiol Aging, 2012. **33**(1): p. 210 e1-7.

8. Ohara, T., et al., *Association study of susceptibility genes for late-onset Alzheimer's disease in the Japanese population.* Psychiatr Genet, 2012. **22**(6): p. 290-3.

9. Lin, Y.L., et al., *Genetic polymorphisms of clusterin gene are associated with a decreased risk of Alzheimer's disease.* Eur J Epidemiol, 2012.

10. Ma, J.F., et al., *Association study of clusterin polymorphism rs11136000 with late onset Alzheimer's disease in Chinese Han population.* Am J Alzheimers Dis Other Demen, 2011. **26**(8): p. 627-30.

11. Liao, Y.C., et al., *ABCA7 gene and the risk of Alzheimer's disease in Han Chinese in Taiwan.* Neurobiol Aging, 2014. **35**(10): p. 2423 e7-2423 e13.

12. Hohman, T.J., et al., *Genetic variation modifies risk for neurodegeneration based on biomarker status.* Front Aging Neurosci, 2014. **6**: p. 183.

13. Tao, Q.Q., et al., *A variant within FGF1 is associated with Alzheimer's disease in the Han Chinese population.* Am J Med Genet B Neuropsychiatr Genet, 2014. **165B**(2): p. 131-6.
