# Supplementary material for: Structural Covariance Network as an Endophenotype in Alzheimer’s Disease-Susceptible Single-Nucleotide Polymorphisms and the Correlations With Cognitive Outcomes
Source: Front Aging Neurosci. 2021 Dec 17;13:721217. doi: 10.3389/fnagi.2021.721217 (PMC8719443; doi:10.3389/fnagi.2021.721217)
Supplement: Supplementary file 2 [file Table_2.docx]

Supplementary Table 2. Linear regression model between network intensity score and cognitive ability screening instrument subdomain scores

|  | | Unstandardized beta; 95% Confidence Interval (p value) | | | | | | | | | | |
| --- | --- | --- | --- | --- | --- | --- | --- | --- | --- | --- | --- | --- |
| Area | | Executive Domains | | | |  | Non-executive Domains | | | | | |
| Index  On  Figure 1 | Network Main Structure | | Attention | Verbal fluency | Abstract thinking | | Mental manipulation |  | Orientation | Long-term memory | Language ability | Drawing |
| A | Basal Ganglia | | -0.227; -0.337~-0.117 (p<0.0001) | -0.180; -0.290~-0.07 (p=0.001) | -0.178; -0.288~-0.068 (p=0.002) | | -0.145; -0.255~-0.036 (p=0.01) |  | -0.188; -0.298~-0.078 (p=0.001) | -0.119; -0.228~-0.009 (p=0.033) | -0.147; -0.257~-0.038 (p=0.009) | -0.115; -0.225~-0.006 (p=0.039) |
| B | Anterior Hippocampus | | 0.366; 0.260~0.471 (p<0.0001) | 0.334; 0.228~0.439 (p<0.0001) | 0.332; 0.226~0.437 (p<0.0001) | | 0.299; 0.194~0.405 (p<0.0001) |  | 0.340;0.235~0.446 (p<0.0001) | 0.271; 0.164~0.377 (p<0.0001) | 0.302; 0.196~0.407 (<0.0001) | 0.266; 0.160~0.373 (p<0.0001) |
| C | Posterior Hippocampus | | 0.447; 0.345~0.549 (p<0.0001) | 0.370; 0.266~0.474 (p<0.0001) | 0.366; 0.262~0.470 (p<0.0001) | | 0.309; 0.203~0.414 (p<0.0001) |  | 0.383; 0.280~0.487 (p<0.0001) | 0.261; 0.155~0.368 (p<0.0001) | 0.312; 0.207~0.417 (p=0.0001) | 0.255; 0.148~0.361; (p<0.0001) |
| D | Right Temporal | | 0.352; 0.246~0.458 (p<0.0001) | 0.313; 0.207~0.419 (p<0.0001) | 0.311; 0.205~0.417 (p<0.0001) | | 0.276; 0.169~0.382 (p<0.0001) |  | 0.321; 0.215~0.427 (p<0.0001) | 0.245; 0.138~0.352 (p<0.0001) | 0.278; 0.171~0.384 (p<0.0001) | 0.241; 0.134~0.348 (p<0.0001) |
| E | Right Thalamus | | 0.249; 0.139~0.359 (p<0.0001) | 0.235; 0.126~0.343 (p<0.0001) | 0.234; 0.125~0.342 (p<0.0001) | | 0.215; 0.107~0.324 (p<0.0001) |  | 0.238; 0.130~0.347 (p<0.0001) | 0.198; 0.09~0.306 (p<0.0001) | 0.217; 0.108~0.325 (p<0.0001) | 0.196; 0.088~0.304 (p<0.0001) |
| F | Left Thalamus | | 0.146; 0.034~0.258 (p=0.011) | 0.106; -0.005~0.217 (p=0.062) | 0.104; -0.0007~0.215 (p=0.066) | | 0.078; -0.032~0.188 (p<0.166) |  | 0.112; 0.001~0.223 (p<0.048) | 0.058; -0.052~0.168 (p=0.303) | 0.08; -0.031~0.190 (p=0.158) | 0.055; -0.055~0.165 (p=0.327) |
| G | Postcentral Gyrus | | 0.052; -0.061~0.165 (p=0.366) | 0.024; -0.088~0.135 (p=0.676) | 0.023; -0.089~0.134 (p=0.689) | | 0.007; -0.104~0.117 (p=0.905) |  | 0.028; -0.084~0.140 (p=0.624) | -0.005; -0.115~0.105 (p=-0.931) | 0.008; -0.103~0.118 (p=0.892) | -0.006; -0.117~0.104 (p=0.909) |
| H | Supplementary Motor Cortex | | -0.194; -0.305~-0.083 (p=0.001) | -0.179; -0.288~-0.069 (p=-0.002) | -0.178; -0.287~-0.068 (p=0.002) | | -0.161; -0.271~-0.052 (p=0.004) |  | -0.182; -0.292~-0.072 (p=0.001) | -0.147; -0.256~-0.038 (p=0.0009) | -0.162; -0272~-0.053 (p=0.004) | -0.144; -0.254~-0.0.35 (p=0.010) |
| I | Inferior Cerebellum | | 0.109; -0.004~0.221 (p=0.058) | 0.110; 0~0.221 (p=0.051) | 0.110; -0.001~0.221 (p=0.051) | | 0.106; -0.004~0.216 (p=0.059) |  | 0.111; 0~0.222 (p=0.05) | 0.101; -0.0009~0.211 (p=0.071) | 0.106; -0.004~0.217 (p=0.058) | 0.1; -0.009~0.210 (p=0.073) |
| J | Lateral Cerebellum | | 0.279; 0.171~0.388 (p=0.0001) | 0.250; 0.141~0.358 (p<0.0001) | 0.248; 0.140~0.356 (p<0.0001) | | 0.221; 0.113~0.329 (p<0.0001) |  | 0.256; 0.147~0.364 (p<0.0001) | 0.197; 0.089~0.305 (p<0.0001) | 0.223; 0.115~0.331 (p=0.0001) | 0.194; 0.086~0.302 (p<0.0001) |
| K | Medial Cerebellum | | 0.186; 0.075~0.297 (p=0.0001) | 0.203; 0.094~0.313 (p<0.0001) | 0.204; 0094~0.313 (p<0.0001) | | 0.203; 0.095~0.312 (p<0.0001) |  | 0.202; 0.093~0.312 (p<0.0001) | 0.199; 0.091~0.307 (p<0.0001) | 0.203; 0.095~0.312 (p<0.0001) | 0.198; 0.090~0.306 (P<0.0001) |
| L | Default Mode Network | | -0.012; -0.125~0.101 (p=0.833) | 0.036; -0.075~0.148 (p=0.521) | 0.038; -0.073~0.149 (p=0.502) | | 0.062; -0.049~0.172 (p=0.272) |  | 0.03; -0.082~0.142 (p=0.6) | 0.078; -0.032~0.188 (p=0.165) | 0.061; -0.05~0.171 (p=0.282) | 0.08; -0.03~0.190 (p=0.154) |
| M | Dorsal Attention Network | | 0.076; -0.037~0.188 (p=0.188) | 0.075; -0.037~0.186 (p=0.187) | 0.075; -0.037~0.186 (p=0.188) | | 0.071; -0.04~0.181 (p=0.21) |  | 0.075; -0.036~0.187 (p=0.185) | 0.066; -0.044~0.176 (p=0.236) | 0.071; -0.04~0.181 (p=0.208) | 0.066 (-0.044~0.176), p=0.240 |
| N | Medial Cerebellum | | 0.129; 0.017~0.241 (p=0.024) | 0.120; 0.01~0.231 (p=0.033) | 0.120; 0.009~0.231 (p=0.034) | | 0.110; 0~0.22 (p=0.051) |  | 0.122; 0.011~0.233 (p=0.031) | 0.1; -0.009~0.210 (p=0.073) | 0.110; 0-0.220 (p=0.05) | 0.099; -0.011~0.209 (p=0.077) |
| O | Cingulate Network | | 0.042; -0.071~0.155 (p=0.467) | 0.073; -0.039~0.184 (p=0.199) | 0.074; -0.038~0.185 (p=0/193) | | 0.087; -0.024~0.197 (p=0.124) |  | 0.069; -0.043~0.180 (p=0.225) | 0.049; -0.016~0.204 (p=0.092) | 0.086; -0.024~0.196 (p=0.127) | 0.095; -0.016~0.205 (p=0.089) |

Data of network intensity scores were Z transformed with cognitive test scores as outcome measures, adjusted for age and educational years
