# Supplementary figures and images for: Structural Covariance Network as an Endophenotype in Alzheimer’s Disease-Susceptible Single-Nucleotide Polymorphisms and the Correlations With Cognitive Outcomes
Source: Front Aging Neurosci. 2021 Dec 17;13:721217. doi: 10.3389/fnagi.2021.721217 (PMC8719443; doi:10.3389/fnagi.2021.721217)

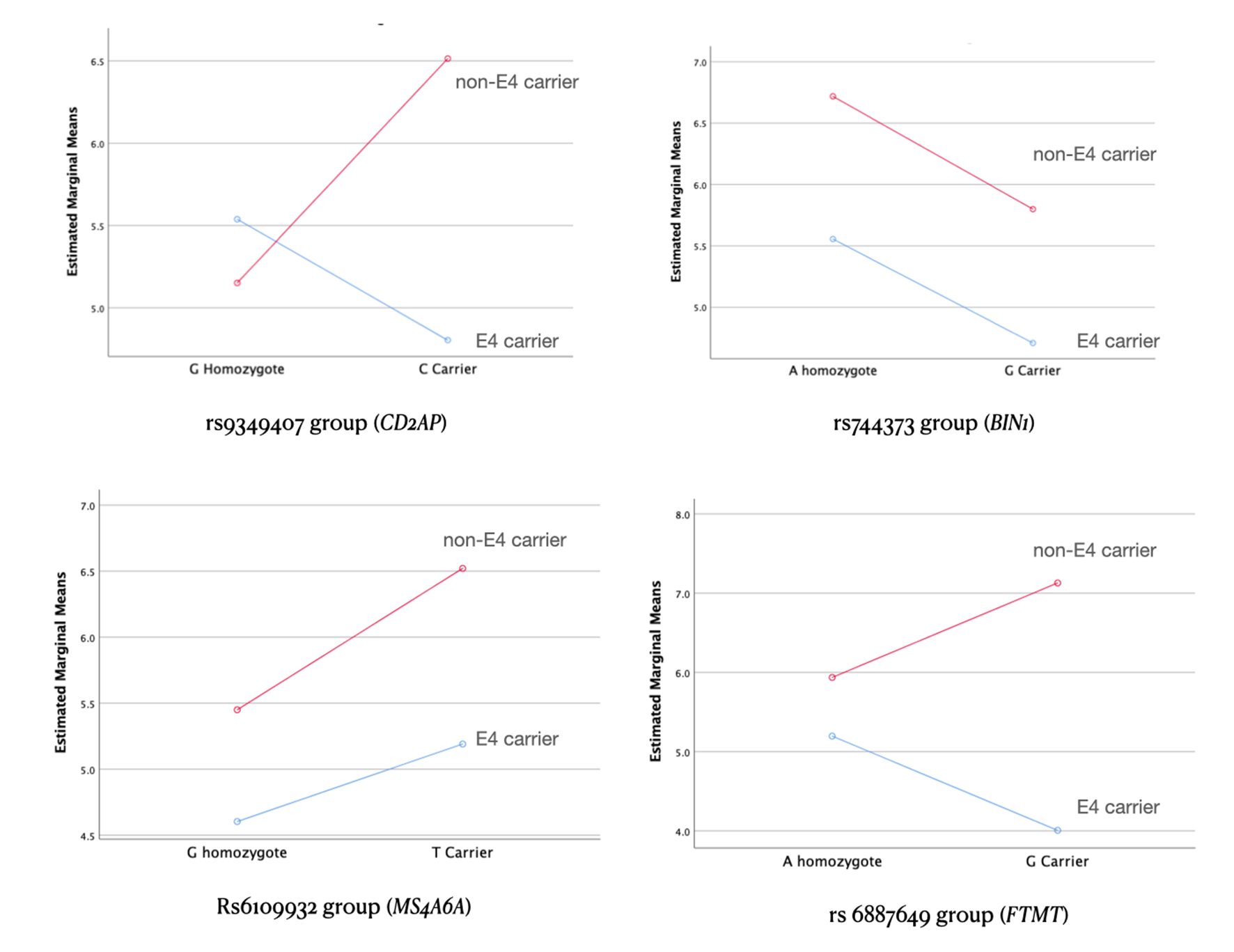

Supplement: Supplementary Figure 1 — Effect of interactions with the ApoE4 gene on STM scores was found in CD2AP, BIN 1, MS4A6A, and FTMT. [file Image_1.tiff]
